# Clinical analysis of abnormal hemoglobin detection results in northern Guangxi, China

**DOI:** 10.1016/j.ymgmr.2026.101371

**Published:** 2026-10-20

**Authors:** Weijia Yang, Xiangjun Wu, Qingyang Xie, Qingping Kang

**Affiliations:** a Department of Eugenics and Genetics, Guilin People's Hospital, Guilin 541002, Guangxi, China; b Department of Clinical Laboratory, Nanning Women and Children's Hospital, Nanning 530003, Guangxi, China; c Department of Pediatrics, Hezhou Guangji Hospital, Hezhou 542827, Guangxi, China

**Keywords:** Globin gene, Abnormal hemoglobin, Hemoglobin electrophoresis, Thalassemia, Phenotype

## Abstract

**Objective:**

To characterize the epidemiological profile, variant spectrum, and clinical phenotypes of abnormal hemoglobins in the northern Guangxi region of China, and to establish and validate a tiered diagnostic strategy combining capillary electrophoresis with multi-platform molecular techniques, thereby providing evidence for regional prevention and control of hemoglobinopathies.

**Methods:**

A retrospective study was conducted. A total of 3890 individuals of reproductive age who visited Guilin People's Hospital between March 2022 and March 2025 underwent initial screening by capillary electrophoresis. Samples confirmed to carry true hemoglobin variants were subjected to genotyping using reverse dot blot hybridization, Sanger sequencing, and third-generation sequencing (TGS). The electrophoretic characteristics, hematological parameters, and genotype–phenotype correlations of different globin variants were analyzed.

**Results:**

The overall detection rate of abnormal hemoglobins was 1.13% (44/3890), encompassing three α-globin and six β-globin variants. HbE was the predominant variant (0.46%), followed by Hb New York. Rare variants, including HbO-Arab and Hb Barcelona, were also identified. The compound variant -α^4.2^α^Q-Thailand^/--^SEA^ manifested as a typical HbH disease phenotype. Double heterozygotes for HbE and β-thalassemia frequently presented with moderate or severe thalassemia requiring long-term clinical intervention. Most simple heterozygotes of β-globin variants exhibited essentially normal hematological parameters. Due to their unaltered net molecular charge, electrophoretically silent variants such as Hb Hekinan II were challenging to detect by conventional capillary electrophoresis, posing a risk of missed diagnosis.

**Conclusion:**

Abnormal hemoglobins in the northern Guangxi region of China display a distinct variant spectrum dominated by HbE. Capillary electrophoresis is suitable for large-scale population screening, and its integration with multi-tiered molecular testing compensates for the limitations of electrophoretic methods, enabling precise identification of rare and silent variants. Implementing genetic counseling and prenatal diagnosis for high-risk populations is a crucial measure to reduce the incidence of severe hemoglobinopathies.

## Highlights

• This is the first report in nearly a decade on abnormal hemoglobinopathies in local reproductive-age women and their spouses.
• The study found that HbE is the most common abnormal hemoglobinopathy locally.
• Extremely rare variants HbO-Arab and Hb Barcelona were identified in the present work.
• HbO-Arab/HbS compound heterozygosity may result in thalassemia intermedia and requires adequate genetic counseling.

## 1 Introduction

Hemoglobinopathy is a group of hereditary monogenic diseases caused by abnormalities in the globin gene.They can be categorized in two principal groups: structural hemoglobinopathies, characterized by abnormal hemoglobin's functionality, and thalassemias, resulting from inadequate.

synthesis of globin chains.These genetic disorders are typically inherited in an autosomal recessive fashion [1], [2]. Hemoglobinopathies are characterized by structural abnormalities of the globin chains. Carriers of certain hemoglobin variants are asymptomatic and non-anemic, e.g., Hb J-Bangkok. Some variants cause polycythemia due to increased oxygen affinity, e.g., Hb New York. Variants with reduced molecular stability and impaired oxygen-carrying capacity can induce hemolytic anemia, e.g., HbS. Other variants cause cyanosis due to oxidation of heme iron to methemoglobin, e.g., HbM. In thalassemia, homozygous or compound heterozygous individuals may present with varying degrees of anemia, including thalassemia major (TM, e.g., genotype βCD41-42/βCD17) and thalassemia intermedia (TI, e.g., genotype β-28/β-28).

As documented in the monograph, more than 1400 types of abnormal hemoglobin variants have been identified to date, with significant regional and ethnic variations [2]. Meanwhile more than 1550 Hb variants are reported in the database. They could lead to Hb polymerization, Hb instability, altered oxygen affinity and decreased oxygen-carrying capacity of Hb or have no clinical manifestations [3], [4]. Located in southern China, Guilin, Guangxi, increased population mobility has led to a diversification of abnormal hemoglobin types in specific regions [2]. Comprehensive genetic screening for hemoglobin variants is crucial for prevention and treatment of these conditions [4]. This study analyzed α- and β-globin variants identified by capillary electrophoresis screening among couples of childbearing age at Guilin People's Hospital, Guangxi, aiming to preliminarily investigate the detection rate of abnormal hemoglobin variants in northern Guangxi and the utility of sequencing techniques for variant confirmation, thereby providing guidance for improving reproductive health and birth outcomes.

## 2 Materials and methods

### 2.1 Inclusion criteria

This retrospective study enrolled a total of 3890 individuals of reproductive age, including pregnant women undergoing antenatal screening and their spouses, at Guilin People's Hospital from March 2022 to March 2025. The participants ranged in age from 17 to 45 years, comprising 1826 males and 2064 females. Individuals were included as study subjects if they tested positive for true hemoglobin variants upon repeat capillary electrophoresis confirmation and did not exhibit characteristic bands of α-thalassemia (e.g., HbCS, HbQS, HbWS, HbH, etc.).

### 2.2 Exclusion criteria

(1) Individuals with characteristic bands of α-thalassemia (e.g., HbCS, HbQS, HbWS, HbH, etc.) detected on the electrophoretogram;
(2) Individuals confirmed to have isolated iron deficiency anemia (serum ferritin <30 ng/mL) without any abnormal hemoglobin variant;
(3) Individuals with incomplete clinical data.

### 2.3 Informed consent

This study was approved by the Ethics Committee of Guilin People's Hospital (approval numbers 2022-072KY, 2023-120KY)·In accordance with the international ethical principles outlined in the WMA “Declaration of Helsinki” (2013) and the CIOMS “International Ethical Guidelines for Biomedical Research Involving Human Subjects” (2002).Before undergoing genetic testing, individuals who tested positive for true abnormal hemoglobin variants upon repeat capillary electrophoresis confirmation were fully informed of the study purpose, procedures, potential benefits, risks, discomforts, and their relevant rights, after which they signed informed consent forms. For participants under 18 years of age, consent was obtained from their legal guardians.

### 2.4 Laboratory methods

#### 2.4.1 Routine blood parameter analysis

Complete blood count (CBC) was performed by using an automated modular blood and body fluid analyzer (Model: XN-10[B3]).

#### 2.4.2 Hemoglobin electrophoresis detection and analysis

Hemoglobin quantification was performed on Sebia Capillarys 2 FlexPiercing capillary electrophoresis platform with manufacturer-provided alkaline buffer (pH 9.4 ± 0.1) and detection wavelength fixed at 415 nm; automated in-tube hemolysis, sample injection and capillary elution/washing were executed following factory preset protocols.

Electropherograms were categorized by the instrument-built Z partition system: Z1 corresponded to the cathodal origin and Z15 to the most anodal end, with increasing electrophoretic mobility from Z1 toward Z15. The horizontal axis represented relative migration distance and the vertical axis indicated absorbance at 415 nm, and peak-area quantification was completed via Phoresis software. HbA was localized in Z9 with moderate mobility; HbA₂ was detected in Z3 with slower migration than HbA; HbF was distributed in Z7, migrating between HbA₂and HbA.

Reference ranges for healthy adults: HbA≥94.5%, HbA_2_ 2.4%–3.5%, HbF≤2.0%. Profiles only presenting qualified peaks within Z3/Z7/Z9 and meeting quantitative criteria were defined as normal.

All extra peaks outside Z3/Z7/Z9 were defined as abnormal bands. α-thalassemia-specific peaks included HbCS in Z1, HbQS/HbWS at the anodal flank of Z9, and fast-moving HbH in Z15; specimens carrying any of these peaks were excluded from variant analysis.

Criteria for validation of abnormal peaks: all samples with unidentified abnormal peaks in initial electrophoresis were retested with residual original EDTA blood. Peaks reproducible at identical Z positions upon repeat electrophoresis were confirmed as true hemoglobin variants; sporadic peaks disappearing in duplicate detection were regarded as denaturation artifacts and excluded.

After removing α-thalassemia-related cases, remaining reproducible abnormal peaks were enrolled as genuine abnormal hemoglobin variants.

#### 2.4.3 Serum ferritin (SF) detection

Serum ferritin (SF) levels were measured using an automated chemiluminescence immunoassay analyzer (Model: Cobas 8000 e801). Samples with SF < 30 ng/mL were assessed to exclude iron deficiency anemia.

#### 2.4.4 Confirmation tests

Abnormal hemoglobin variants were confirmed by molecular biology methods. HbE was confirmed using reverse dot blot hybridization polymerase chain reaction (RDB-PCR), The remaining abnormal hemoglobin variants were validated by Sanger sequencing. Variants that tested negative by Sanger sequencing or remained unidentifiable were further analyzed by third-generation sequencing (TGS).

##### 2.4.4.1 Reverse dot blot hybridization (RDB-PCR)

**Primers:** Oligonucleotides with defined base sequences were labeled with biotin at the 5′ end to amplify target DNA fragments encompassing all loci of interest.

**Probes:** Nineteen probes were used, including 41-42M, 654M, -28M, 71-72M, 17M, βEM, 14-15M, 27/28M, 31M, 37M, 43M, -29M, -30M, -32M, -50M/N, CAPM, IntM, IVS-I-1M, and IVS-I-5M.

**Amplification Instrument:** Bio-Rad T100 Thermal Cycler.

**Hybridization Instrument:** Yaneng YN-H16 Constant-Temperature Hybridization Incubator.

**Result Interpretation (using HbE as an example):** The presence of abnormal HbE was determined by the appearance of blue spot signals at the test loci. The blank control showed no coloration at any spot on the membrane. A negative result was defined as coloration at all normal (N) spots and no coloration at any mutation (M) spots on the membrane. A positive result indicating an HbE heterozygote was defined as coloration at both the normal (N) spot and the corresponding mutation (M) spot. An HbE homozygote was defined as no coloration at the normal (N) spot but coloration at the mutation (M) spot.

##### 2.4.4.2 Sanger sequencing

**Primers:** Two HBB amplicons were designed. Fragment 1 spanned from c.-273 to c.316**-**16 (1100 bp), covering the regulatory region, exon 1, and introns 1–2. Fragment 2 extended from c.316‐654 to the 3′ polyA region (800 bp), covering exon 3.

**Instrument/Platform:** ABI PRISM 3730xl DNA Analyzer (Applied Biosystems, USA).

**Software and Tools for Gene Sequence Analysis:** Bioinformatics analysis was performed using the Indigo online tool (https://www.gear-genomics.com/indigo/). Teal was used to trim low-quality chromatogram traces and verify bidirectional sequencing consistency. Indigo was used to split heterozygous alleles and align sequences to the reference genome to identify single nucleotide variants (SNVs) and insertions/deletions (InDels). False positives were filtered out through quality control and annotation against dbSNP and ClinVar databases. All chromatograms were manually reviewed, and all variants were validated by bidirectional sequencing.

γ-globin (HBG) gene sequencing:

Sanger dideoxy chain termination sequencing was used to detect mutations in the γ-globin gene promoter regions. Gene-specific primers were designed for the promoter regions of both the Gγ-globin gene (HBG2) and the Aγ-globin gene (HBG1), amplifying fragments of 676 bp and 674 bp, respectively. Primer sequences and product lengths are provided in the supplementary materials. Sequencing results were analyzed using Chromas software, and the chromatograms were compared against the normal HBG1 and HBG2 reference sequences from GenBank (accession numbers: GU324925.1 and GU324926.1) and the human reference genome GRCh38 to identify promoter region mutations.

##### 2.4.4.3 Third-generation sequencing (TGS)

**Primers:** Genomic DNA was amplified by PCR using primers covering the majority of known structural variants, single nucleotide variants, and insertions/deletions within the HBA1, HBA2, and HBB genes.

**HBB Gene Sequencing Region:** The targeted region spanned chr11:5223857–5,235,806 (wild-type). Gap primers were additionally used to cover various β-globin gene deletions of different extents.

**Targeted Gene Sequencing:** TGS covered the β- and δ-globin genes.

**Instrument/Platform**: PacBio Sequel II platform using circular consensus sequencing (CCS) mode.

**Software and Tools for Sequence Analysis**: Processed sequencing reads were aligned to the hg38 reference genome using pbmn2 (Pacific Biosciences). Structural variants were identified based on the HbVar, Ithanet, and LOVD databases. Haplotype analysis was performed using pb amplicon clustering software and WhatsHap (version 0.18). Single nucleotide variants (SNVs) and insertions/deletions (indels) were identified using FreeBayes (version 1.3.4).

### 2.5 Denominator flow

**Figure:**
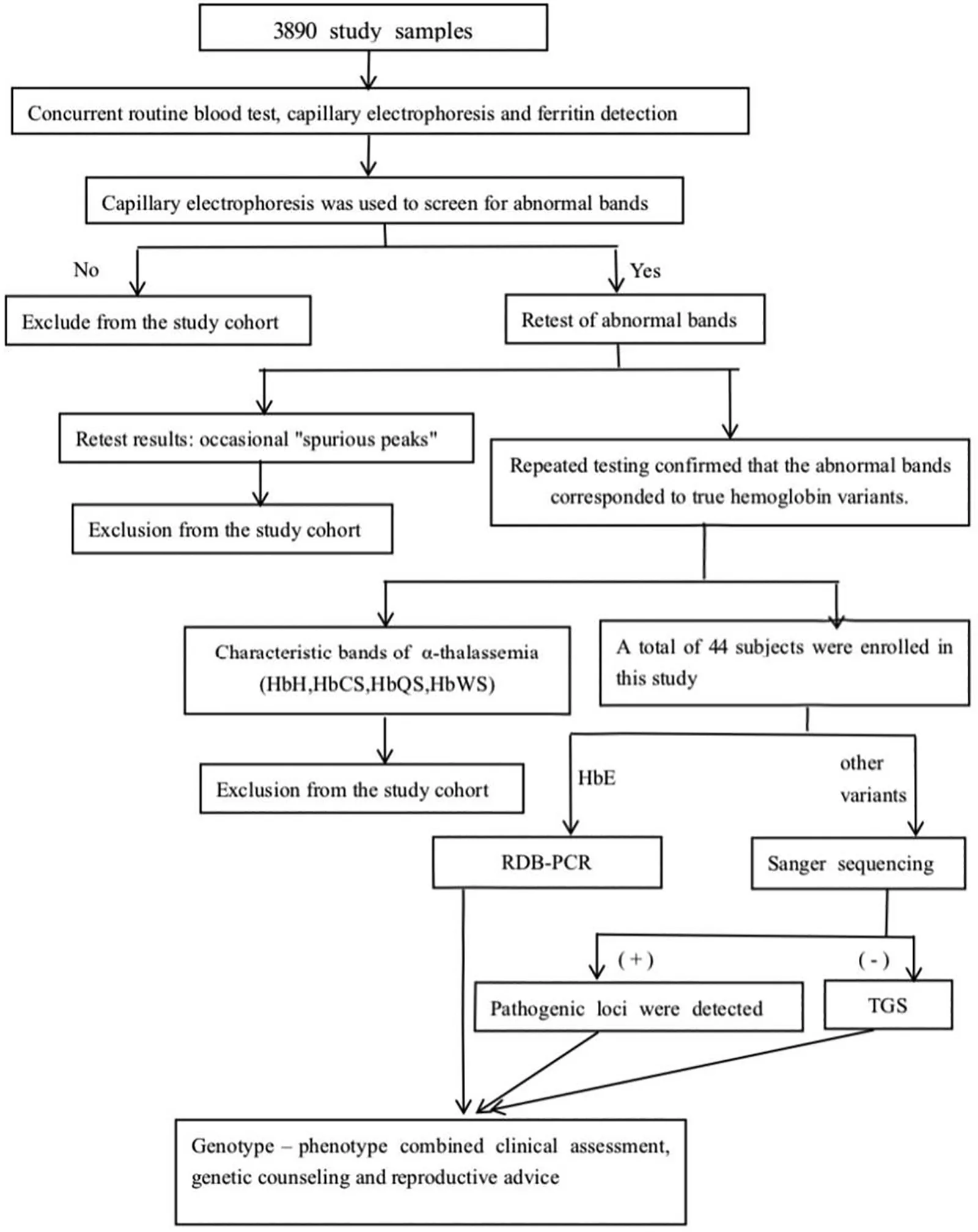

### 2.6 Statistical analysis

Count data were expressed as percentages (%). Means and standard deviations were calculated using SPSS 30.0 software. As no inter-group comparisons were involved, hypothesis testing or related statistical tests were not applied.

## 3 Results

A total of 44 cases of abnormal hemoglobin were detected among the 3890 samples, representing a detection rate of 1.13% and comprising 9 distinct types**,**including 3 types on the α-globin gene cluster and 6 types on the β-globin gene cluster. The detection rate of the HbE carrier state was 0.46% (18/3890), which was the highest among rare variants, followed by Hb New York at 0.33% (13/3890).Of note, these detection rates were based on the population undergoing prenatal and pregnancy screening at our hospital and do not represent the true prevalence in the general local population.

### 3.1 Detection of α-globin variants

Table 1 lists 2 cases of -α^4.2^α^Q-Thailand^/αα(HBA1: c.223G > C, het)(cases 1,2, case 1 shown in Fig. 1A, B), 1 case of -α^4.2^α^Q-Thailand^/-α ^3.7^(HBA1: c.223G > C, het)f, 1 case of -α^4.2^α ^Q-Thailand^/--^SEA^(HBA1: c.223G > C, het) (case 4, shown in Fig. 1C, D), the detection rate for carriers of -α^4.2^α^Q-Thailand^/αα was 0.10% (4/3890), 1 case of αα/αα Hb G-Honolulu (HBA2: c.91 G > C, het) (case 5, shown in Fig. 1E, F), and 1 case of αα/αα Hb Hekinan II(HBA1: c.84G > T, het) combined with Gγ-158C > T, Aγ-158C > T (het, case 6, shown in Fig. 1G, H, I), the detection rate for each of the latter two variants was 0.03% (1/3890).

**Table 1:** Hematological and Capillary Electrophoresis Characteristics of α-Globin Variants.

Genotype	No.	n	Sex	Age (year)	Hb (g/L)	MCV (fL)	MCH (pg)	HbA %	HbA2%	HbF %	Abnormal Hb Variant(%)	Migration position (zone,X-axis)	Parameter Characteristics
-α^4.2^α^Q-Thailand^/αα (HBA1: c.223G > C,het)	1	1	Female	37	111	77.6	26.1	69.1	1.9	Variant	28.3	Z(F), 185	Microcytic hypochromic
-α^4.2^α^Q-Thailand^/αα (HBA1: c.223G > C,het)	2	1	Female	28	114	78.5	25.2	67.7	1.8	Variant	29.8	Z(F), 185	Microcytic hypochromic
−α^4.2^α^Q-Thailand^ /−α 3.7 (HBA1: c.223G > C,het)	3	1	Male	30	137	72.1	21.6	56.4	1.1	Variant	41.7	Z(F), 185	Microcytic hypochromic
-α^4.2^α^Q-Thailand^/--SEA (HBA1: c.223G > C,het)	4	1	Female	27	79	54.5	16.4	0	1.5	Variant	92.1	Z(F), 185	Microcytic hypochromic
Hb G-Honolulu； (HBA2: c.91G > C,het)	5	1	Male	36	159	91	31	74.4	1.2	0	24	Z(D), 205	Zonenormocytic normochromic
Hb HekinanII(HBA1:c.84G > T,het) Gγ-158C > T， Aγ-158C > T	6	1	Female	32	124	84.5	30	86.5	2.2	11.3	Not detected	Not detected	reduced HbA2, significantly elevated HbF

Footnotes Defining: Hb (hemoglobin), MCV (mean corpuscular volume), MCH (mean corpuscular hemoglobin), HbA (hemoglobin A), HbA₂ (hemoglobin A2), HbF (fetal hemoglobin), het(heterozygous).

Reference ranges: Hb (110–150 g/L), MCV (82–100 fL), MCH (27–34 pg), HbA (≥ 94.5%), HbA₂ (2.4–3.5%), HbF (< 2%)

**Fig. 1:**
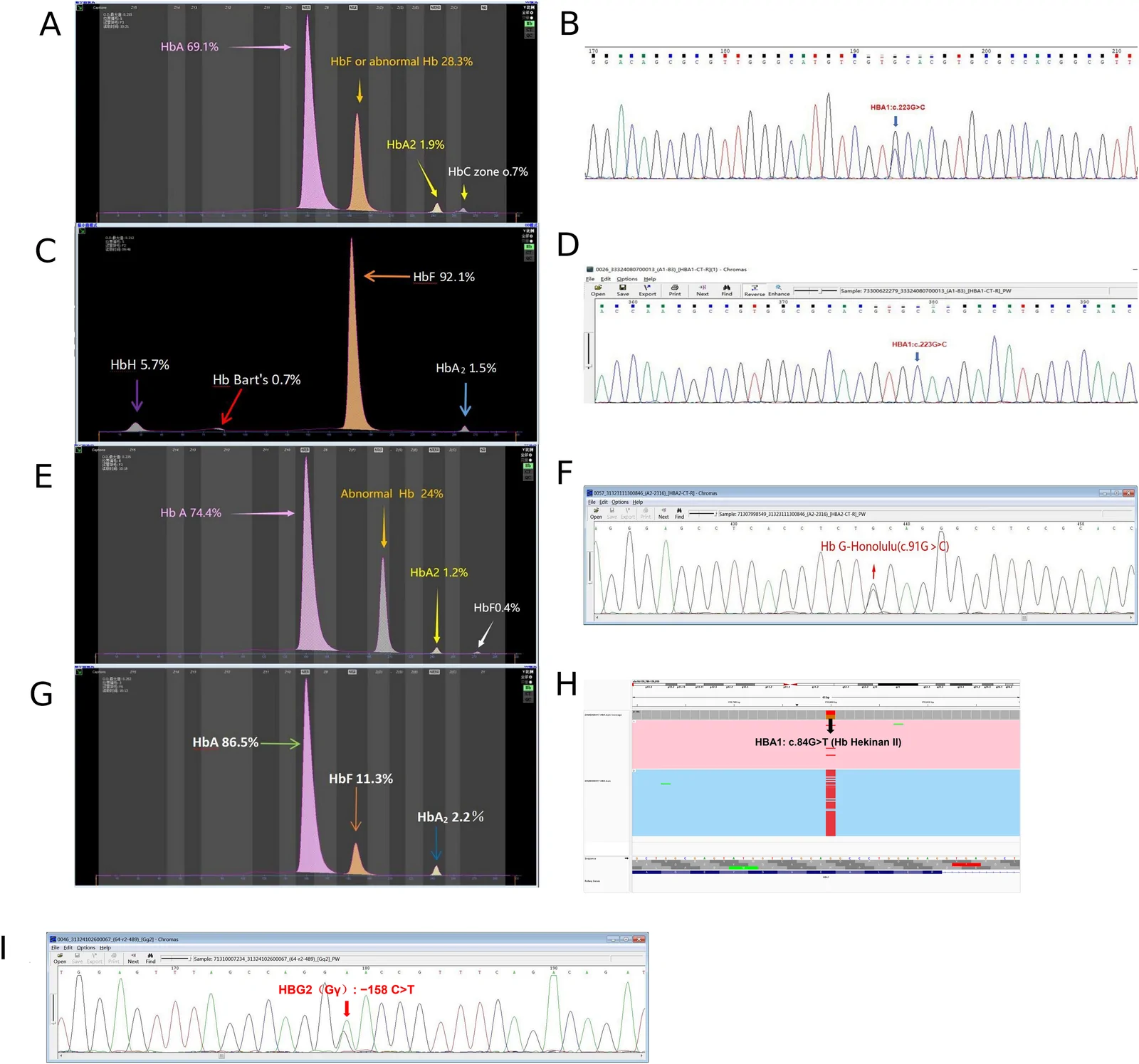
:Capillary electropherograms and corresponding sequencing chromatograms of four cases. **1A, Case 1:** capillary electropherogramof -α^4.2^α^Q-Thailand^/αα (HBA1: c.223G > C, het), showing abnormal hemoglobin peaks in zones F and C. **1B, Case 1:** Sanger sequencing validation of -α^4.2^α^Q-Thailand^/αα (HBA1: c.223G > C, het). The arrow indicates the double peak at c.223G > C. **1C, Case 4:** capillary electropherogram of -α^4.2^α^Q-Thailand^/--^SEA^ (HBA1: c.223G > C, het), showing abnormal hemoglobin peaks in zones F, Z15, and Z12. **1D, Case 4:** Sanger sequencing validation of -α^4.2^α^Q-Thailand^/--^SEA^ (HBA1: c.223G > C, het). The arrow indicates the double peak at c.223G > C. **1E, Case 5:** capillary electropherogram of Hb G-Honolulu (HBA2: c.91G > C, het), showing abnormal hemoglobin peaks in zones D and Z1. **1F, Case 5:** Sanger sequencing validation of Hb G-Honolulu (HBA2: c.91G > C, het). The arrow indicates the double peak at c.91G > C. **1G, Case 6:** capillary electropherogram of Hb Hekinan II (HBA1: c.84G > T, het) compounded with Gγ-158C > T and Aγ-158C > T. **1H, Case 6:** third-generation sequencing (TGS) alignment view of the HBA1 gene, showing the heterozygous c.84G > T mutation (Hb HekinanII). The arrow indicates the specific mutation site within the aligned reads. **1I, Case 6:** sanger sequencing chromatogram of the HBG2 (Gγ-globin) gene promoter region, confirming the heterozygous −158C > T mutation. Gene-specific primers for HBG2 were used to distinguish it from HBG1 (Aγ-globin). The arrow indicates the double peak at the specific mutation site.

### 3.2 Detection of β-globin variants

Table 2 lists 6 types: the most common was abnormal HbE (HBB: c.79G > A，het) with 16 cases (cases 7–22, case 7 shown in Fig. 2A, B), 2 cases of abnormal HbE combined with β-thalassemia heterozygosity (case 23 shown in Fig. 2C, D, case 24 shown in Fig. 2E, F).The detection rate for carrying HbE was 0.46% (18/3890),Next was Hb New York (HBB: c.341 T > A，het) with 13 cases (case 25 shown in Fig. 2G, H), the detection rate was 0.33%(13/3890),Hb J-Bangkok (HBB: c.170 G > A，het) with 4 cases (case 38 shown in Fig. 3A, B),the detection rate was 0.10%(4/3890), Hb G-Taipei (HBB: c.68 A > G，het) (case 42 shown in Fig. 3C, D), HbO-Arab (HBB: c.364G > A，het) (case 43 shown in Fig. 3E, F), and Hb Barcelona (HBB: c.283G > C，het) (case 44 shown in Fig. 3G, H) with 1 case each, the detection rates for the subsequent three variants were all 0.03% (1/3890).

**Table 2:** Hematological and Capillary Electrophoresis Characteristics of β-Globin Variants.

Genotype	No.	n	Sex	Age (year)	Hb (g/L)	MCV (fL)	MCH (pg)	HbA%	HbA2%	HbF %	Abnormal Hb Variant (%)	Migration position (zone,X-axis)	Parameter Characteristics
βN/βE(HBB:c.79G > A，het)	7–22	16	Male(5) Female(11)	28.13 ± 4.08	128.44 ± 14.97	77.03 ± 5.66	24.75 + 3.01	72.39 ± 3.26	3.33 ± 0.31	0.19 ± 0.33	24.23 ± 3.18	Z(E) ，227.8 ± 0.6	Microcytic hypochromic
βIVS-II-654/βE (HBB:c.79G > A,het)	23	1	Male	17	65	70.8	20.4	18.1	4.6	33.4	43.9	Z(E) ，227	Microcytic hypochromic
βCD41–42/βE (HBB:c.79G > A,het)	24	1	Male	22	48	68.8	19	4.1	6.2	44.1	45.6	Z(E) ，227	Microcytic hypochromic
Hb New York(HBB:c.341 T > A ,het)	25- 37	13	Male(10) Female(3)	35.31 ± 6.05	142.69 ± 15.84	89.46 ± 6.03	29.94 ± 2.12	53.9 ± 4.29	2.75 ± 0.78	0.29 ± 0.63	43.05 ± 3.96	Z11，121.9 ± 0.9	Zonenormocytic normochromic
Hb J-Bangkok(HBB:c.170G > A,het)	38- 41	4	Female	30 ± 5.48	117.25 ± 10.37	85.73 ± 14.21	27.48 ± 5.84	46.6 ± 0.83	2.38 ± 0.28	0.05 ± 0.10	50.98 ± 0.76	Z12，93.0 ± 0.7	Zonenormocytic normochromic
HbG-Taipei(HBB:c.68 A > G,het)	42	1	Male	45	147	79	31	54.6	2.7	0	42.7	Z(D), 199	No typical microcytic hypochromic
HbO-Arab(HBB:c.364G > A,het)	43	1	Female	28	122	84	29.9	55.9	Unknown	5	39.1	Z(A2), 241	Zonenormocytic normochromic
HbBarcelona， (HBB:c.283G > C,het)	44	1	Female	28	129	89.3	30.0	62.2	3.0	Variant	34.8	Z(F), 182	Zonenormocytic normochromic

Footnotes Defining:Hb(hemoglobin),MCV(mean corpuscular volume),MCH(mean corpuscular hemoglobin),HbA(hemoglobin A)HbA 2(hemoglobin A2). HbF(fetal hemoglobin),het(heterozygous).

Reference ranges: Hb (110–150 g/L), MCV (82–100 fL), MCH (27–34 pg), HbA (≥ 94.5%), HbA₂ (2.4–3.5%), HbF (< 2%)

**Fig. 2:**
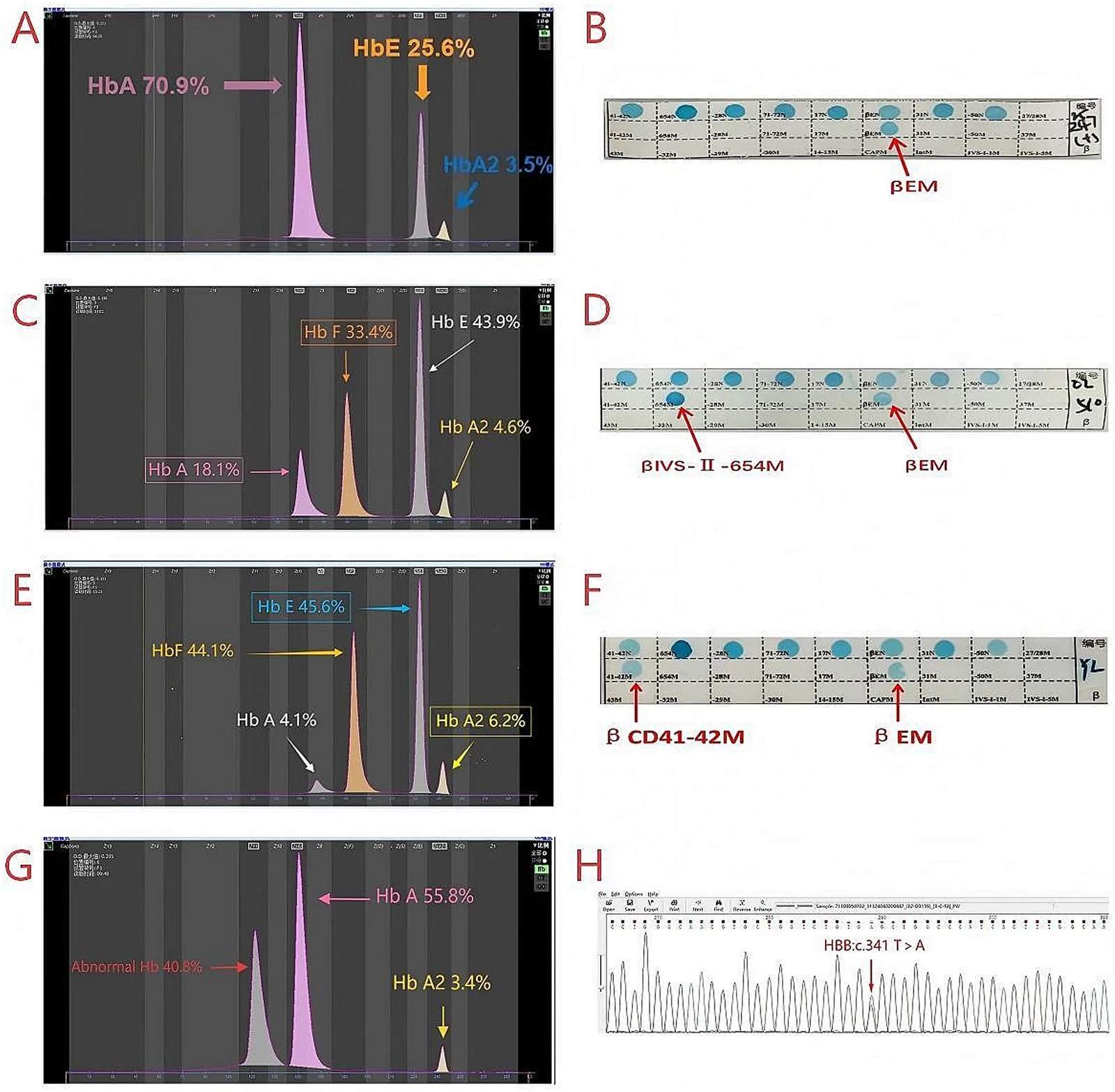
Capillary electropherograms and corresponding strip blots / sequencing chromatograms of four cases. **2A, Case 7:** capillary electropherogram of βN/βE (HBB: c.79G > A, het), showing abnormal hemoglobin peaks in zone E. **2B, Case 7:** reverse dot blot hybridization strip of βN/βE (HBB: c.79G > A, het). The arrow indicates the positive βE mutation site. **2C, Case 23:** capillary electropherogram of βIVS-II-654/βE (HBB: c.79G > A, het), showing abnormal hemoglobin peaks in zone E. **2D, Case 23:** reverse dot blot hybridization strip of βIVS-II-654/βE (HBB: c.79G > A, het). Arrows indicate the positive βIVS-II-654 M and βE mutation sites. **2E, Case 24:** capillary electropherogram of βCD41–42/βE (HBB: c.79G > A, het), showing abnormal hemoglobin peaks in zone E. **2F, Case 24:** reverse dot blot hybridization strip of βCD41–42/βE (HBB: c.79G > A, het). Arrows indicate the positive βCD41–42 M and βE mutation sites. **2G, Case 25:** capillary electropherogram of Hb New York (HBB: c.341 T > A, het), showing abnormal hemoglobin peaks in zone Z11. **2H, Case 25:** Sanger sequencing validation of Hb New York (HBB: c.341 T > A, het). The arrow indicates the double peak at c.341 T > A.

**Fig. 3:**
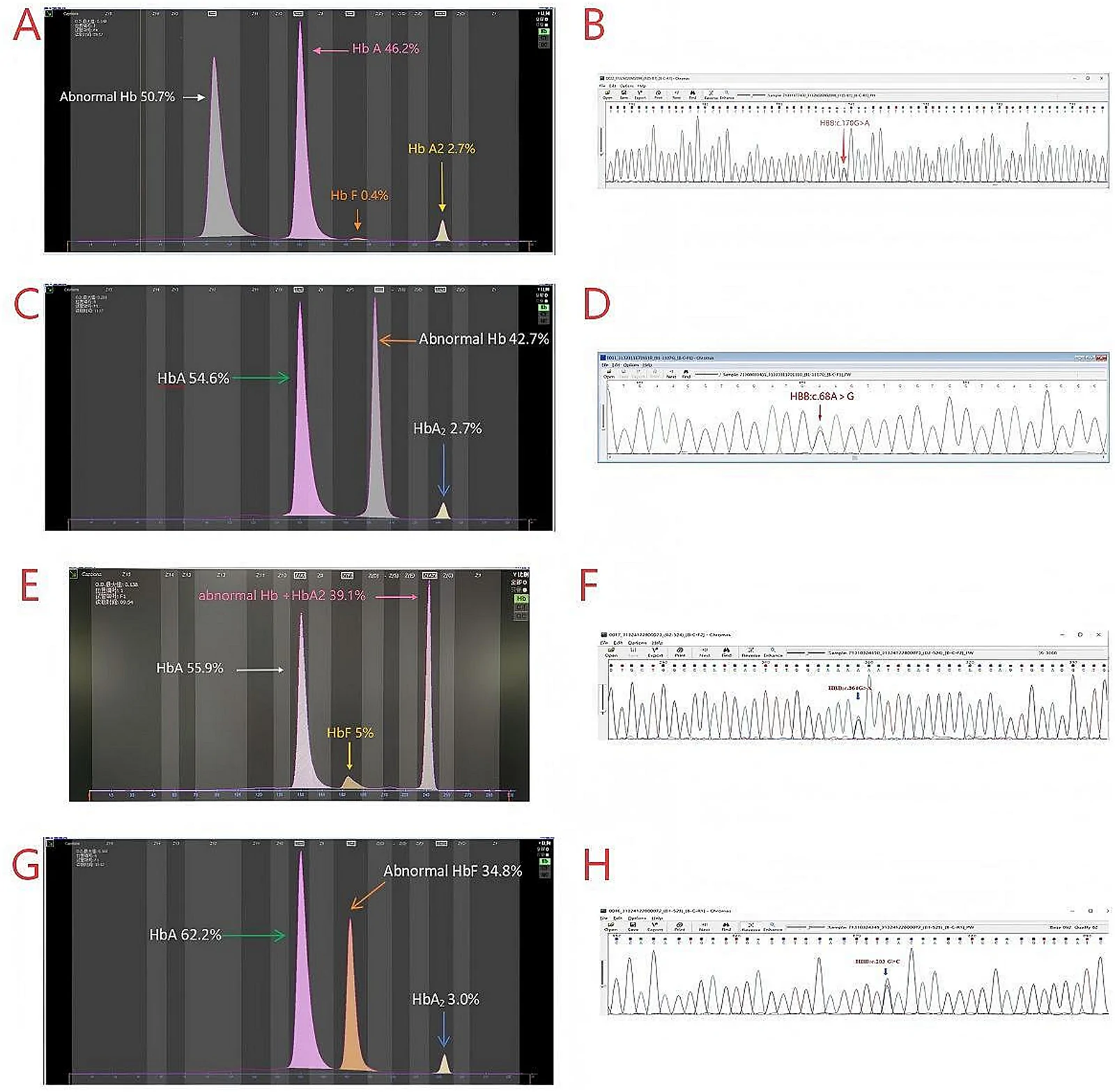
Capillary electropherograms and corresponding sequencing chromatograms of four cases. **3A, Case 38:** capillary electropherogram of Hb J-Bangkok (HBB: c.170G > A, het), showing abnormal hemoglobin peaks in zone Z12. **3B, Case 38:** Sanger sequencing validation of Hb J-Bangkok (HBB: c.170G > A, het). The arrow indicates the double peak at c.170G > A. **3C, Case 42:** capillary electropherogram of HbG-Taipei (HBB: c.68 A > G, het), showing abnormal hemoglobin peaks in zone D. **3D, Case 42:** Sanger sequencing validation of HbG-Taipei (HBB: c.68 A > G, het). The arrow indicates the double peak at c.68 A > G. **3E, Case 43:** capillary electropherogram of HbO-Arab (HBB: c.364G > A, het), showing the abnormal hemoglobin peak overlapping with the HbA2 peak, with a combined quantitation of 39.1%. **3F, Case 43:** Sanger sequencing validation of HbO-Arab (HBB: c.364G > A, het). The arrow indicates the double peak at c.364G > A. **3G, Case 44:** capillary electropherogram of Hb Barcelona (HBB: c.283G > C, het), showing abnormal hemoglobin peaks in zone F. **3H, Case 44:** Sanger sequencing validation of Hb Barcelona (HBB: c.283G > C, het). The arrow indicates the double peak at c.283G > C.

## 4 Discussion

### 4.1 α-Globin chain variants

**Hb^Q-Thailand^ (HBA1: c.223G** **>** **C)** results from a mutation at codon 74 of the α1-globin gene (GAC → CAC), replacing aspartic acid with histidine. Hb^Q-Thailand^ is typically linked with -α^4.2^ deletion thalassemia, often asymptomatic and easily misdiagnosed as a simple -α^4.2^ deletion. Previous studies [5] reported Hb^Q-Thailand^ as the most common Hb variant in Shaoguan, Guangdong (0.17%, 17/10,285), mainly among the Hakka population in Meizhou, Guangdong, and widely distributed in Southeast Asia, including Taiwan, Thailand, and Singapore due to Hakka migration. This study detected 2 cases of **-α**^**4.2**^**α**^**Q-Thailand**^**/αα**with no anemia, microcytic hypochromic features, normal or low HbA2, and abnormal bands in the F and C zones on capillary electrophoresis (Fig. 1A), consistent with multiple studies [6], [7], [8], [9]. However, when **-α**^**4.2**^**α**^**Q-Thailand**^**/αα** is combined with large deletions like –SEA, it leads to HbH disease, with no HbA band on capillary electrophoresis and abnormal bands in the F, Z15 (HbH), and Z12 (Hb Bart's) zones (Fig. 1C). Individuals with the-α^4.2^α^Q-Thailand^/--^SEA^ genotype completely lose the ability to synthesize normal α-globin chains, resulting in the absence of HbA. The excess β and γ chains form HbH and Hb Bart's, respectively. Meanwhile, the abnormal hemoglobin formed by the mutated αQ chain binding with γ chains migrates to zone F.Literature [7]suggests that HbH-4.2^Q-Thailand^ has slightly more severe anemia than HbH-WS, approaching deletion-type HbH (e.g., −α^4.2^/--^SEA^). Hb-Westmead is a mildly unstable hemoglobin variant resulting from a CAC → CAG mutation at codon 122 of the α2-globin gene (CD122, CAC > CAG). Clinically, individuals with HbH-WS are either asymptomatic or present with mild anemia. When mild anemia occurs, it is mainly observed in pregnant women and is attributed to pregnancy-related anemia rather than the HbH-WS heterozygote state itself, consistent with the phenotype of silent α-thalassemia.Southeast Asian deletion-type α-thalassemia (--^SEA^) is the most common deletional α-thalassemia in southern China. Heterozygous carriers of this deletion are clinically asymptomatic, have normal or mildly decreased hemoglobin levels, and exhibit reduced MCV and MCH. When --^SEA^ (α0-thalassemia) coexists with an α + −thalassemia allele, the condition is termed hemoglobin H (HbH) disease. This genotype typically involves defects in three out of four α-globin genes, leading to reduced α-chain synthesis and an excess of β-chains. The resulting imbalance between α- and β-chains damages red blood cells and causes chronic hemolytic anemia [10].In the study by Jiang F,this is evidenced by the finding that none of the 32 individuals with a coinherited Hb Westmead and α0-thal presented with a Hb H band on electrophoresis, suggesting that the a/ βchain balance is only mildly impaired, and not enough redundant β-globin chains are left to form Hb H(β4) [11].This explains the mechanism underlying the mild clinical phenotype of HbH-WS resulting from compound heterozygosity of HbH-WS and the Southeast Asian deletion (--^SEA^).In this study, a pregnant woman with -α^4.2^ Hb^Q-Thailand^/--^SEA^, excluding iron deficiency anemia, had an Hb of only 79 g/L,this is consistent with the report by Liang KL et al. [10].

**Hb G-Honolulu (HbA2: c.91 G** **>** **C)** results from a glutamine-to-glutamic acid substitution at codon 31 of the α-globin gene, altering the retention time of hemoglobin subcomponents in cation-exchange high-performance liquid chromatography (HPLC). Previous studies [12] noted that hemoglobinopathies interfere with HbA1c measurements, particularly in cation-exchange HPLC, where an “E window” appeared instead of HbA1c, with an area smaller than typical HbE variants (25.2% vs. 30–35%), indicating another variant, confirmed as Hb G-Honolulu. In this study, the abnormal Hb band appeared in the D zone (Fig. 1E), with no anemia or microcytic hypochromic features, consistent with reports by Lin et al. [7], Yang et al. [9], and Luo et al. [13], a decreased HbA2 level was also observed, which is consistent with the reports by Lin et al. [7] and Luo et al. [13].

**Hb Hekinan II (HBA1: c.84G** **>** **T)**, first identified in Japanese populations in 1988, results from a codon 27 mutation (GAG→GAT) in the α1-globin gene, converting glutamic acid to aspartic acid, eliminating a heme restriction enzyme site. Due to the lack of charge change, this variant is detectable by HPLC but not by capillary or cellulose electrophoresis, leading to potential misdiagnosis.Although Hb Hekinan (α27 Glu → Asp) has no net charge change compared with Hb A, cation-exchange high-performance liquid chromatography (HPLC) can still effectively separate this variant. Unlike traditional electrophoresis, which primarily responds to the overall molecular charge, cation-exchange HPLC is more sensitive to the spatial distribution of surface charges and the stereochemical properties of amino acid side chains. The substitution of glutamic acid (four‑carbon side chain) with aspartic acid (three‑carbon side chain) shortens the side chain at position α27, thereby altering the local electrostatic potential and conformational microenvironment around the mutation site. Under conditions of a weak cation-exchange column (e.g., PolyCAT A coated with polyaspartic acid) combined with pH and salt double-gradient elution, these subtle physicochemical differences are amplified, resulting in measurable retention time differences, typically appearing as a small shoulder peak or a partially resolved peak before the main Hb A peak. This explains why the electrophoretically silent Hb Hekinan can be presumptively identified by cation-exchange HPLC [14], [15], [16]. In this study, no abnormal bands were detected by capillary electrophoresis; however, the patient exhibited decreased HbA2 and elevated HbF levels. Sanger sequencing identified Gγ-158C > T and Aγ-158C > T mutations (Fig. 1I), which are located in the promoter regions of the γ-globin genes (HBG2 and HBG1). These mutations disrupt the binding of transcriptional repressors, leading to increased γ-globin expression and HbF synthesis, thereby explaining the elevated HbF level. To further investigate the cause of the decreased HbA2 level, third-generation sequencing (TGS) was performed. Using long-read alignment at the single-molecule level, TGS successfully distinguished the highly homologous HBA1 and HBA2 genes and identified an Hb Hekinan II variant (HBA1: c.84G > T) in the HBA1 gene (Fig. 1H). Therefore, the decreased HbA2 level in this case is attributable to reduced synthesis of the α1-globin chain.Previous studies [7], [13], [17] reported no anemia, possible microcytic hypochromic features, and reduced HbA2 in simple Hb Hekinan. Chen Xingyuan [18] reported a family with Hb Hekinan combined with αα/--SEA showing no hematological changes or significant anemia, suggesting that α1-globin gene mutations are compensated by stronger α2-globin synthesis. In this case, Hb Hekinan II with Gγ-158C > T, Aγ-158C > T showed significantly elevated HbF, no anemia, or microcytic hypochromic features, but low HbA2, with no prior literature on this compound type.

### 4.2 β-Globin variants

Hb E (HBB: c.79G > A) is the most common hemoglobinopathy in Southeast Asia, caused by a point mutation at codon 26 (GAG→AAG) in the β-globin gene, replacing glutamic acid with lysine. This mutation in exon 1 creates an abnormal splice site, reducing β-globin production and causing β + thalassemia [19]. HbE prevalence reaches 40%–50% in northeastern Thailand [20]. HbE heterozygotes typically have no anemia but show microcytic hypochromic features, with abnormal HbE bands in the E zone on electrophoresis. HbE interaction with β-thalassemia can lead to HbE/β-thalassemia, confirmed by RDB-PCR, often denoted as βEM (βCD26: GAG→AAG). HbE/β-thalassemia shows significant clinical heterogeneity, ranging from mild to severe anemia (Hb 25–133 g/L) [19], [21], with elevated HbF and reduced or absent HbA. β0/HbE is generally more severe than β+/HbE, with lower HbA. In this study, case 23 (βIVS-II-654/βE, β+/HbE) had Hb 65 g/L, requiring transfusion every 40 days; case 24 (βCD41–42/βE, β0/HbE) had Hb 48 g/L, requiring transfusion every 30 days. In Southeast Asia, particularly Thailand and Malaysia, HbE detection, especially in couples planning pregnancy, warrants prenatal diagnosis if one partner carries HbE and the other carries β-thalassemia. Urza Bhattarai et al. [22]. reported a 53-year-old Nepalese male with a 15-year history of abdominal distension, multiple transfusions, hepatosplenomegaly, hyperbilirubinemia, and gallstones, with Hb 67 g/L, HPLC showing HbA2 + E 79%, HbF 9.5%, HbA 6.5%, later confirmed as homozygous HbE in both partners. Jayasree et al. analyzed 84 homozygous HbE patients, finding varied severity, with approximately one-third having splenomegaly and 28.0% having hyperbilirubinemia. Couples with HbE heterozygosity should undergo genetic counseling and, if necessary, further molecular testing.The carrier rate of HbE heterozygotes is relatively high in this region, warranting increased clinical awareness. If one partner is identified as carrying abnormal HbE, and the other partner is found to have either β-thalassemia or an abnormal HbE variant upon screening, the couple should receive genetic counseling, confirmatory testing by RDB-PCR, and reproductive guidance.

**Hb New York (HBB: c.341** **T** **>** **A)** is a hemoglobin variant [β113(G15) Val → Glu, GTG > GAG], where hydrophobic valine at position 113 is replaced by acidic glutamic acid, affecting molecular hydrophobicity and reducing stability compared to HbA, making it prone to degradation. It is relatively common, with normal hematological parameters, no anemia, and electrophoresis showing bands in the Z11 zone (Fig. 2G). This study detected 43.05 ± 3.96% abnormal Hb in heterozygotes, consistent with multiple studies [5], [6], [8], [9], [23], [24].

**Hb J-Bangkok (HBB: c.170 G** **>** **A**) results from a codon 56 mutation (GGC → GAC, aspartic acid to glycine), altering β-globin conformation. It is the third most common after HbE and Hb New York. Clinically similar, Hb J-Bangkok shows abnormal bands in the Z12 zone with HbA2 on electrophoresis (Fig. 3A). This study detected 50.98 ± 0.76% abnormal Hb in 4 heterozygous cases, consistent with studies [5], [6], [8], [9], [13]. Hb J-Bangkok combined with different thalassemia types shows varied phenotypes. Li Yumin et al. [25]. reported that Hb J-Bangkok with α-thalassemia resembles α-thalassemia phenotypes, while with β-thalassemia, hematological changes depend on the β-thalassemia type. In Southeast Asia, Hb J-Bangkok is common, with Wanicha Tepakhan et al. [26] reporting two cases of Hb J-Bangkok with β + thalassemia (IVS-1-5 G > C), showing mild anemia (Hb 101 and 106 g/L), requiring further large-scale studies.

**Hb G-Taipei (HBB: c.68 A** **>** **G)**, a codon 22 point mutation (Glu → Gly), shows prolonged retention time than HbAO on HPLC HbA1c analysis due to increased positive charge, elevating HbA1c levels [27]. In this study, the heterozygous Hb G-Taipei showed 42.7% abnormal Hb in the D zone (Fig. 3C), with no anemia and normal hematological parameters, consistent with studies [5], [9], [28]. A study [29] reported a 28-year-old male with Hb G-Taipei and Hb Lepore-Boston heterozygosity, showing reduced MCV and MCH but no anemia due to compensatory erythropoiesis. Hb G-Taipei with thalassemia or other variants requires case-specific analysis.

**HbO-Arab (HBB: c.364G** **>** **A),** extremely rare in this region, is a β-globin gene defect (β121 Glu → Lys), common in the Balkans and Middle East. HbO heterozygotes typically have normal Hb levels [30].In Case 33, capillary electrophoresis revealed an abnormally high peak in the A2 region, which was attributed to overlapping of the abnormal hemoglobin with HbA2 (Fig. 3E). Miniar Kalai et al. [30]. reported that homozygous HbO-Arab is associated with mild to moderate anemia, while HbO/β-thalassemia compound heterozygotes show severe anemia. A case of HbO-Arab with IVS-I-1 (G > A), a common β0-thalassemia mutation in Tunisians, was reported. Van Gammeren et al. [31]. reported a newborn with HbO-Arab (HBB: c.364G > A) and Hb D-Los Angeles (HBB: c.664G > C), showing mild microcytic anemia at one-year follow-up. Elbashir et al. [32]. reported 13 African-American children and adults with Hb S/O, aged 2.7 to 62.5 years, all with hemolytic anemia (median Hb 8.7 g/dL, range 6.1–9.9 g/dL), and severe sickle hemoglobinopathy complications, including gallstones, nephropathy, leg ulcers, stroke, and osteomyelitis. HbO-Arab with other Hb variants or β-thalassemia shows complex and diverse phenotypes, generally more severe than the previous three variants.

**Hb Barcelona (HBB: c.283G** **>** **C)** results from a substitution of aspartic acid with histidine at position 94 of the β-globin chain, extremely rare. Initially identified in a Spanish family with mild erythrocytosis by Aguilar i Bascompte JL et al. [33], the increased oxygen affinity of Hb Barcelona is explained by the disruption of a salt bridge between β94-Asp and β146-His. Phillips SE et al. [34]. confirmed a doubled oxygen affinity compared to HbA. This explains why carriers typically show erythrocytosis without anemia. In this study, 34.8% abnormal Hb was detected (Fig. 3G), close to the 37% reported by Phillips SE et al. [26].Hb Barcelona was detected for the first time in this region, and relevant literature reports are also very limited.

## 5 Limitations

This study has several limitations. First, the samples were derived exclusively from a specific patient population at Guilin People's Hospital; therefore, the observed detection rate of abnormal hemoglobins (1.13%) may not fully represent the true prevalence in the general population of northern Guangxi, China. Second, as a retrospective, single-center study, some rare variants (e.g., Hb Barcelona) were identified in only one case each. The assessment of genotype–phenotype associations for these rare variants requires validation through larger sample sizes and multicenter studies. Third, electrophoretically silent variants such as Hb Hekinan II carry a high risk of being missed during routine capillary electrophoresis screening. The widespread adoption of sequencing and other molecular methods for definitive diagnosis represents a current trend. Future studies should expand the screening scope and conduct prospective cohort studies to provide more robust data for the precise prevention and control of abnormal hemoglobin disorders in high-prevalence regions.

## 6 Conclusion

This study is the first to establish and apply a tiered screening strategy combining capillary electrophoresis with Sanger sequencing and third-generation sequencing (TGS) in Guilin, Guangxi, China, and systematically elucidates the variant spectrum and hematological and phenotypic characteristics of abnormal hemoglobin disorders in this region. Most abnormal hemoglobin variants exhibit typical electrophoretic features and can be rapidly screened by capillary electrophoresis; however, electrophoretically silent rare variants such as Hb Hekinan II are prone to being missed by routine electrophoresis. This study challenges traditional clinical understanding by demonstrating that abnormal hemoglobin disorders are not necessarily accompanied by microcytic hypochromic changes, and some cases may present with normal or even elevated red blood cell indices, providing an important basis for differential diagnosis from classical thalassemia. The findings emphasize that individuals with the -α^4.2^α^Q-Thailand^/--^SEA^ compound variant, which manifests as a typical HbH disease phenotype, require close clinical follow-up, whereas those with HbE compounded with β-thalassemia need clinical intervention. For high-risk carriers and at-risk couples, enhanced genetic counseling and prenatal diagnosis are essential to effectively prevent the occurrence of severe hemoglobinopathies. This study supplements population-based data on abnormal hemoglobin disorders in northern Guangxi, China, and provides reliable data references and practical evidence for regional hemoglobinopathy screening, clinical differential diagnosis, and birth defect prevention.

## Availability of data and material

All data generated or analyzed during this study are included in this article. Further enquiries can be directed to the corresponding author.

## Publication consent

We confirm that all authors of this manuscript have reviewed and consented to its submission and publication.

## CRediT authorship contribution statement

**Weijia Yang:** Writing – review & editing, Resources, Project administration, Methodology, Funding acquisition, Conceptualization. **Xiangjun Wu:** Writing – original draft, Validation, Investigation, Data curation. **Qingyang Xie:** Writing – review & editing, Supervision, Formal analysis, Data curation. **Qingping Kang:** Validation, Software, Investigation, Data curation.

## Ethical approval

The study was conducted in accordance with the Declaration of Helsinki(as was revised in 2013). The study was approved by Ethics Committee of the Guilin People's Hospital (Approval number: 2023-120KY).

## Funding source

1) Open Project of NHC Key Laboratory of Thalassemia Medicine.

Project Title: Precise Diagnosis and Intervention Strategies for Thalassemia with Genotype-Phenotype Inconsistency.

Grant Number: GJWJWDP202303.

2) Self-funded Project of Western Medicine Category, Health Commission of Guangxi Zhuang Autonomous Region.

Project Title: Genotyping Study of Silent and Rare Variants of Thalassemia in Couples Undergoing Pregnancy.

Grant Number: *Z*-C20221640.

## Declaration of competing interest

The authors declare that they have no known competing financial interests or personal relationships that could have appeared to influence the work reported in this paper.

## Data Availability

The data that has been used is confidential.

## Appendix A Supplementary data

Supplementary material 1: Primer sequences and PCR product sizes for HBG1 and HBG2 promoter region amplification.
