# Supplementary material for: Clinical analysis of abnormal hemoglobin detection results in northern Guangxi, China
Source: Mol Genet Metab Rep. 2026 Oct 20;49:101371. doi: 10.1016/j.ymgmr.2026.101371 (PMC13717739; doi:10.1016/j.ymgmr.2026.101371)
Supplement: Supplementary file 1 [file mmc1.docx]

**Table. Primer Sequences for HBG1 and HBG2 Promoter Region Amplification**

| Primer | Primer Sequence (5'-3') | Product (bp) |
| --- | --- | --- |
| G1 (Forward) | GCACTGAAACTGTTGCTTTATAGGAT | 676 |
| G2 (Reverse) | GGCGTCTGGACTAGGAGCTTATTG |  |
| A1 (Forward) | GCGCTGAAACTGTGGCTTTAT | 674 |
| G2 (Reverse) * | GGCGTCTGGACTAGGAGCTTATTG |  |

*Note: G2 serves as the shared reverse primer for both Aγ and Gγ amplification.
